# The Protective Mechanism of Moderate Intensity Continuous Training on TMAO-Induced Myocardial Injury Based on NMR Metabolomics

**DOI:** 10.3390/ijms26188902

**Published:** 2025-09-12

**Authors:** Hong Zou, Lijing Gong, Caihua Huang, Donghai Lin, Yimin Zhang

**Affiliations:** 1 Department of Physical Education, Xiamen University, Xiamen 361005, China; zou_hong0402@126.com; 2 Key Laboratory of Exercise and Physical Fitness of Ministry of Education, Beijing Sport University, Beijing 100084, China; lijing.gong@bsu.edu.cn; 3 China Institute of Sport and Health Science, Beijing Sport University, Beijing 100084, China; 4 Research and Communication Center of Exercise and Health, Xiamen University of Technology, Xiamen 361024, China; caihua.huang@foxmail.com; 5 Key Laboratory for Chemical Biology of Fujian Province, MOE Key Laboratory of Spectrochemical Analysis & Instrumentation, College of Chemistry and Chemical Engineering, Xiamen University, Xiamen 361005, China

**Keywords:** NMR metabolomics, MICT, TMAO, heart, protective mechanism

## Abstract

The purpose of this study was to explore the protective effect of 8 weeks of Moderate Intensity Continuous Training (MICT) on TMAO-induced myocardial injury in mice and its metabolic regulatory mechanism based on nuclear magnetic resonance (NMR) metabolomics methods. Male C57BL/6J mice were randomly allocated into the following groups: Control group (Con, n = 15), TMAO-induced myocardial injury group (TMAO, n = 15), and TMAO-induced with MICT intervention group (Exe, n = 15). TMAO and Exe groups underwent 8 weeks of high-dose TMAO gavage to establish a myocardial injury model, with the Exe group additionally receiving 8 weeks of MICT intervention (60 min/session, 5 sessions/week, 50% MRC). After the 8 weeks of interventions, the mouse heart function was tested using cardiac ultrasound equipment; myocardial histology was evaluated using HE staining; and myocardial tissue samples were collected for NMR metabolomics analysis. Compared with the Con group, the HR in the TMAO group was significantly increased, while EF and LVFS were significantly decreased. Compared with the TMAO group, the HR in the Exe group was significantly reduced, and EF and LVFS were significantly increased; NMR metabolomics analysis showed that, compared with the Con group, five metabolic pathways including phenylalanine metabolism, tyrosine metabolism, and TCA cycle were significantly altered in the TMAO group; compared with the TMAO group, ten metabolic pathways related to amino acid metabolism (such as alanine, glycine, etc.), energy metabolism (TCA cycle), and oxidative stress (purine metabolism) were significantly regulated in the Exe group. MICT could effectively alleviate TMAO-induced myocardial injury in mice by regulating multiple targets within the myocardial metabolic pathways. These findings provide a theoretical basis for the clinical application of exercise intervention in myocardial injury treatment.

## 1. Introduction

Cardiovascular diseases (CVDs), characterized by their high prevalence and mortality rates, constitute a significant threat to human health. According to data reported by the World Health Organization (WHO, 2019) [1], CVDs remain the leading cause of death among chronic non-communicable diseases worldwide. At present, although a variety of drugs and medical technologies are available for treating CVDs, their efficacy in reducing morbidity and mortality is still restricted. Hence, actively exploring and developing more effective prevention and treatment strategies is of vital importance for the control and management of cardiovascular diseases.

Recent studies have unveiled the significant role of metabolites secreted by gut microbiota in the development of cardiovascular diseases [2,3,4]. Of particular concern is Trimethylamine-N-Oxide (TMAO), a metabolite generated through the combined action of gut microbiota and hepatic flavin monooxygenase. It has been widely recognized as closely associated with the prognosis of CVDs and plays a crucial role in the process of atherosclerosis [5,6,7]. By employing metabolomics techniques, researchers have identified a clear dose-dependent relationship between TMAO concentration in the blood and the risk of CVDs, which for the first time links TMAO to the risk of CVDs [8]. In animal experimental models, TMAO has been confirmed to directly induce atherosclerosis and thrombosis [9,10,11], although its specific mechanisms require further investigation. Moreover, TMAO is also involved in regulating various metabolic pathways in the body and is associated with chronic diseases such as fatty liver, chronic kidney disease, and heart failure [12,13,14]. The heart is a metabolically active organ, and its function is closely related to the healthy status of the body’s metabolic state [15]. Therefore, elucidating the mechanisms by which TMAO regulates cardiac metabolism is of great significance for revealing the pathogenesis of TMAO-related cardiac diseases and may also provide a direction for the development of new therapeutic strategies for cardiac diseases.

Exercise is not only an effective non-pharmacological treatment and prevention method, but also has numerous benefits for overall health promotion. Evidence shows that exercise can improve cardiometabolic risk factors, enhance cardiovascular system function, reduce the risk of arrhythmia, and alleviate vascular responses during coronary artery ischemia–reperfusion [16,17,18,19]. In particular, Moderate Intensity Continuous Training (MICT) has been proven to be highly effective in preventing the occurrence of various chronic diseases. Studies indicate that aerobic exercise can enhance physical fitness, improve myocardial function and antioxidant capacity, and help normalize myocardial metabolism [20]. Swimming has been shown to improve the left ventricular configuration of hypertensive rats induced by a high-salt diet and alleviate symptoms of heart failure [21]. Despite the well-documented cardioprotective effects of exercise, the understanding of how exercise modulates myocardial injury induced by TMAO, a metabolite produced by gut microbiota, remains limited.

As the end products of gene transcription and translation, metabolites represent the culmination of multiple biological processes and thus provide a snapshot of the current state of a biological system [22]. Given their position at the downstream end of the biological information flow, metabolites integrate the effects of genetic, epigenetic, and environmental factors, making them highly informative indicators of overall physiological status. By combining detection techniques such as NMR, LC-MS, and GC-MS with pattern recognition methods, metabolomics analysis is capable of elucidating metabolic alterations in biological systems in response to external stimuli. As such, it has been extensively employed in the discovery of early disease biomarkers, the investigation of molecular mechanisms underlying disease development, and the exploration of therapeutic mechanisms of both pharmacological and non-pharmacological interventions. The advantages of NMR-based metabolomics lie in its non-destructive and unbiased profiling of complex samples, thereby ensuring the objectivity and reproducibility of the analyses.

The present study employs NMR metabolomics to investigate the metabolic impact of long-term, high-dose TMAO ingestion on myocardial injury in mice, as well as the potential ameliorative mechanisms of MICT, thereby providing novel scientific insights for cardio-protection and disease prevention.

Although ^1^H-NMR provides a non-destructive and reproducible snapshot of high-abundance cardiac and plasma metabolites, its intrinsic detection floor (low-micromolar to high-nanomolar range) inevitably overlooks low-concentration signaling molecules that may be pivotal in TMAO-driven myocardial injury, such as specific oxylipins or transient acyl-carnitines. Spectral crowding and minute pH-, ionic strength-, or matrix-induced chemical-shift variations can further complicate peak assignment and quantification. Consequently, metabolite variations observed in this study should be interpreted as indicators of integrated metabolic stress rather than definitive disease-specific signatures. Complementary LC-MS/GC-MS analyses and isotope-tracer validation will be required to capture the full spectrum of TMAO-related metabolic disturbances [23,24,25].

## 2. Results

### 2.1. The Results of Body Weight, Heart Weight, and Ultrasound Examination

**Figure 1:**
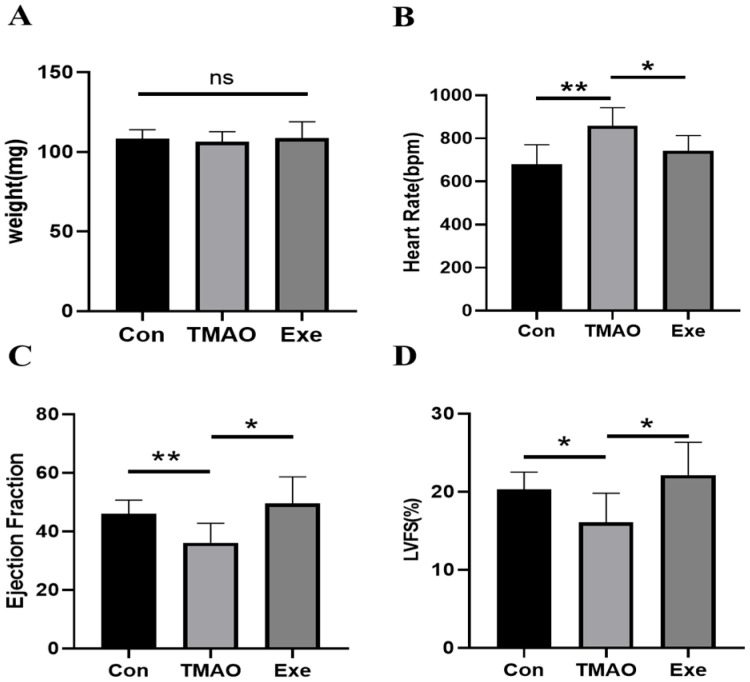
The heart weight and ultrasound examination in the Con, TMAO, and Exe groups of mice. (**A**) The heart weight of mice in three groups. (**B**) The HR of mice in three groups. (**C**) The EF of mice in three groups. (**D**) The LVFS of mice in three groups. Statistical significances: ns: *p* ≥ 0.05; *: *p* < 0.05; **: *p* < 0.01.

Mice in both the TMAO and Exe groups exhibited lower body weight gains than those in the Con group. However, the differences in body weight gains among the three groups were not statistically significant. This indicates that, while TMAO intake and exercise both influence mouse body weight, their effects do not significantly differ (Figure S1). There were also no significant differences in heart weight among the Con, TMAO, and Exe groups of mice (Figure 1A). There were significant differences in HR among the Con, TMAO, and Exe groups. Compared with the Con group, the TMAO group exhibited a significant increase in HR (*p* < 0.01), and the Exe group also showed a significant increase compared with the TMAO group (Figure 1B, *p* < 0.05). Compared to the Con group, the TMAO group exhibited significantly decreased EF (*p* < 0.01) and LVFS (*p* < 0.05), indicating impaired cardiac function. However, MICT significantly improved both parameters (Figure 1C,D, *p* < 0.05), suggesting a cardioprotective effect. These results highlight the potential detrimental impact of chronic high-dose TMAO intake on cardiac performance and underscore the critical role of MICT in cardiac protection.

### 2.2. H&E Staining Results of Myocardial Tissue

**Figure 2:**
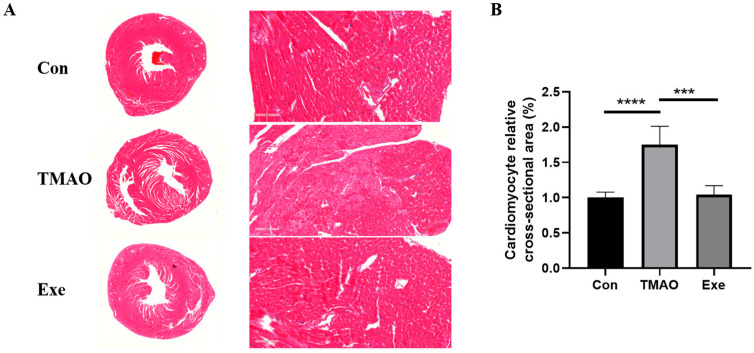
Histopathological analysis of the myocardial tissue in the Con, TMAO, and Exe groups of mice. (**A**) Representative HE staining of the myocardial tissue among groups. (**B**) Column diagram of cardiomyocyte relative cross-sectional area. Data are expressed as mean ± SD. Statistical significances: ***: *p* < 0.001; ****: *p* < 0.0001.

Under the optical microscope, the nuclei of cardiomyocytes appeared bluish brown, while the cytoplasm appeared pink. Comparative analysis revealed that myocardial tissue in the TMAO group showed slight signs of damage compared to the Con group. However, such damage was rarely observed in the myocardial tissue of mice in the Exe group that underwent MICT intervention (Figure 2A). These results indicate that MICT has a significant cardioprotective effect and can alleviate myocardial tissue damage induced by TMAO. Additionally, the statistical analysis of the cross-sectional area (CSA) of myocardial cells showed that TMAO injury resulted in the larger relative CSA, while MICT intervention significantly restored the cardiomyocyte CSA (Figure 2B).

### 2.3. NMR Metabolomics Analysis Results of Aqueous Metabolites in Mouse Myocardial Tissue

#### 2.3.1. NMR Spectra and Metabolite Identification

**Figure 3:**
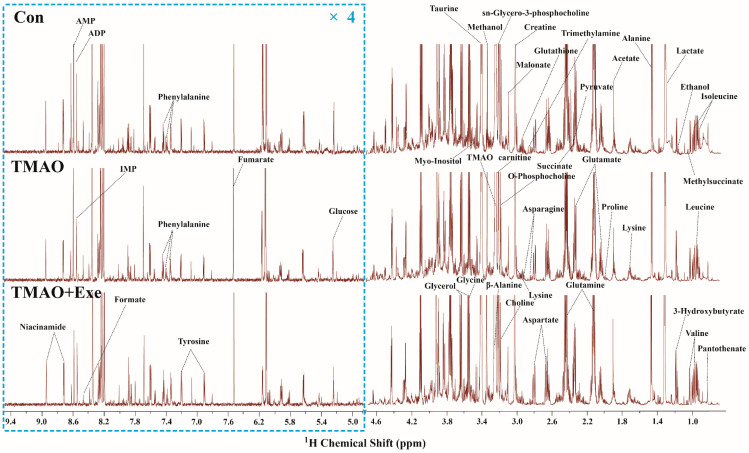
Typical 850 MHz ^1^H NMR spectra recorded on aqueous extracts derived from the myocardial tissue in three groups of mice.

The typical 850 MHz ^1^H NMR spectra of assigned hydrophilic metabolites extracted from the myocardial tissue of mice in the Con, TMAO, and Exe groups are exhibited (Figure 3). To maintain consistency in the vertical scaling, the water peak region (4.8–5.0 ppm) was removed, with the regions of 0.8–4.8 ppm and 5.0–9.4 ppm (within the dashed-line boxes) retained. To enhance spectral clarity, the display ratio of the 5.0–9.4 ppm region was magnified fourfold relative to the 0.8–4.8 ppm region. Metabolite identification in the spectra was performed by integrating information from the published literature, the Chenomx NMR Suite software, and the HMDB database. A total of 43 metabolites were ultimately identified (Table S1).

#### 2.3.2. Multivariate Statistical Analysis for NMR Spectra of Metabolites Extracted from the Myocardial Tissue

**Figure 4:**
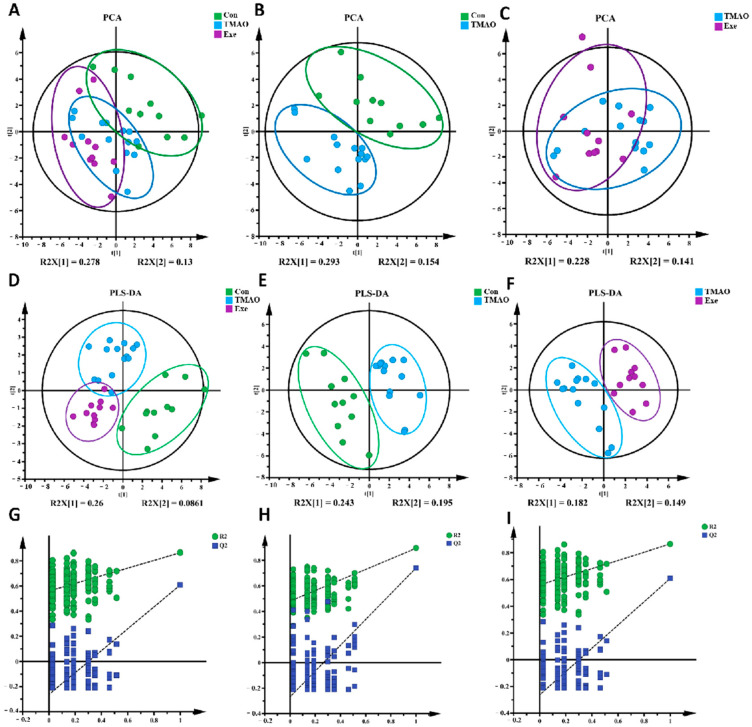
Multivariate analysis of identified metabolites in ^1^H NMR spectra of aqueous extracts derived from the myocardial tissue in three groups of mice. (**A**–**C**) PCA scores plot. (**D**–**F**) PLS-DA scores plot. (**G**–**I**) Cross-validation diagram of PLS-DA model.

To reveal the metabolic characteristics of myocardial tissue in the three groups of mice, a multivariate statistical analysis was performed based on NMR spectra. First, a PCA model was constructed to detect the discrimination trends of different groups’ myocardial metabolic patterns. The PCA score plot (Figure 4A–C) showed obvious differences in myocardial metabolic patterns among the three groups. To further observe the metabolic pattern differences between groups, a supervised PLS-DA model was established. The results revealed distinct separations in the metabolic profiles of the three groups (Figure 4D). The metabolic profile of the TMAO group was clearly distinguished from the Con group along t [1] (Figure 4E), and the Exe group’s profile was distinct from the TMAO group along both t [1] and t [2] (Figure 4F). To ensure the PLS-DA model’s robustness and reliability, cross-validation with 200 random permutations was conducted. The cross-validation plots (Figure 4G–I) showed that the blue regression line of Q^2^ had a negative intercept on the *Y*-axis. Except for the Con vs. TMAO group comparison, the R^2^ and Q^2^ values of the other two group comparisons were significantly higher than the rest when X = 1, confirming the PLS-DA model’s reliability. These results indicate that TMAO induces significant metabolic disorders in myocardial tissue, while MICT can partially reverse this damage, restoring metabolic homeostasis.

#### 2.3.3. Identifications of Important, Differential, and Characteristic Metabolites of Aqueous Extracts Derived from the Myocardial Tissue

**Figure 5:**
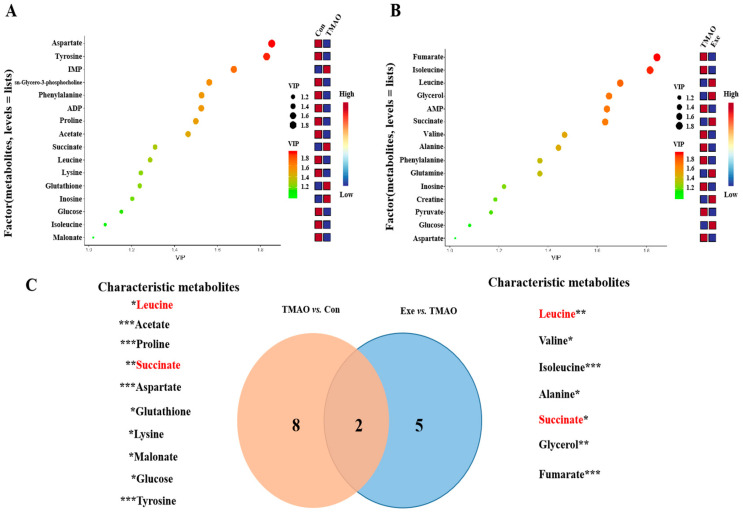
Screening of important and characteristic metabolites in the PLS-DA model of the three groups of the myocardial tissue of mice. (**A**) The important metabolites of the TMAO vs. Con group. (**B**) The important metabolites of the Exe vs. TMAO group. (**C**) Characteristic metabolites. The overlapping metabolites are in red. *: *p* < 0.05; **: *p* < 0.01; ***: *p* < 0.001.

Based on the PLS-DA score plot analysis, 16 and 15 important metabolites (VIP > 1) were identified from TMAO vs. Con and Exe vs. TMAO, respectively (Figure 5A,B). Relative levels of metabolites were calculated from peak intensities (Figure S2). Comparison of TMAO vs. Con and Exe vs. TMAO revealed 10 and 7 significantly changed differential metabolites, respectively (Table S2). Combining VIP and significance (VIP > 1 and *p* < 0.05), a total of 10 characteristic metabolites were found in TMAO vs. Con. Among these, eight metabolites were found to be decreased, which included leucine, acetate, proline, aspartate, lysine, malonate, glucose, and tyrosine. Conversely, two metabolites were increased, namely succinate and glutathione. In the Exe vs. TMAO comparison, a total of seven characteristic metabolites were identified. Specifically, six metabolites were found to be decreased, which are leucine, valine, isoleucine, alanine, glycerol, and fumarate. In contrast, one metabolite was increased, which is succinate. Two common characteristic metabolites, leucine and succinate, were identified in both comparisons (Figure 5C).

#### 2.3.4. Identifications of Significantly Altered Metabolic Pathways of Aqueous Extracts Derived from the Myocardial Tissue of Mice

**Figure 6:**
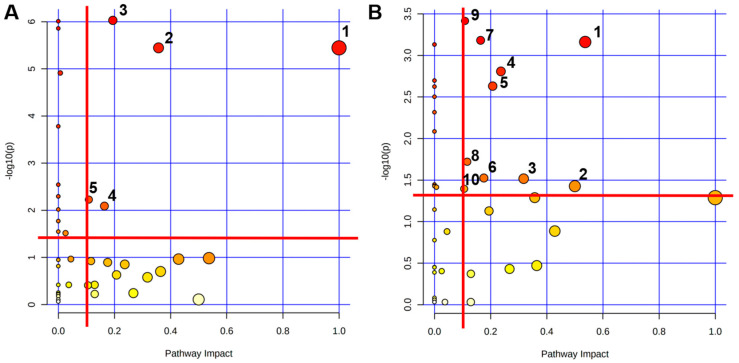
Significantly altered metabolic pathways identified through the pairwise comparisons among three murine cardiac groups. (**A**) TMAO vs. Con group. The significantly altered metabolic pathways were labeled with the following numbers: (1) Phenylalanine, tyrosine, and tryptophan biosynthesis; (2) Phenylalanine metabolism; (3) Nicotinate and nicotinamide metabolism; (4) Tyrosine metabolism; (5) Citrate cycle (TCA cycle). (**B**) Exe vs. TMAO group. The significantly altered metabolic pathways were labeled with the following numbers: (1) Alanine, aspartate, and glutamate metabolism; (2) D-Glutamine and D-Glutamate metabolism; (3) Glycine, serine, and threonine metabolism; (4) Glycerolipid metabolism; (5) Purine metabolism; (6) Arginine and proline metabolism; (7) Tyrosine metabolism; (8) Arginine biosynthesis; (9) TCA cycle; (10) Glyoxylate and dicarboxylate metabolism.

To reveal the mechanisms of TMAO-induced metabolic disruption in mouse myocardial injury and the impact of exercise intervention on these metabolic pathways, the pathway analysis was performed using MetaboAnalyst 5.0 based on the 43 identified metabolites. Using a significance threshold of *p* > 0.05 and PIV > 0.1, our analysis revealed distinct metabolic alterations. Compared to the Con group, the TMAO group exposure significantly disrupted five key pathways (Figure 6A): Phenylalanine, tyrosine, and tryptophan biosynthesis; Phenylalanine metabolism; Nicotinate and nicotinamide metabolism; Tyrosine metabolism and Citrate cycle (TCA cycle). Conversely, Exe vs. TMAO modulated ten compensatory pathways (Figure 6B): Alanine, aspartate, and glutamate metabolism; D-Glutamine and D-Glutamate metabolism; Glycine, serine, and threonine metabolism; Glycerolipid metabolism; Purine metabolism; Arginine and proline metabolism; Tyrosine metabolism; Arginine biosynthesis; TCA cycle; Glyoxylate and dicarboxylate metabolism. These results demonstrate that MICT counteracts TMAO-induced cardiotoxicity through multi-pathway metabolic reprogramming toward a cardioprotective phenotype.

## 3. Discussion

Gut microbiota and their metabolites play crucial roles in maintaining systemic health by regulating diverse metabolic processes [26,27]. Dietary phosphatidylcholine, abundantly found in fish, red meat, shellfish, and eggs, is converted to TMAO via gut microbial deacylation. Recent studies confirm TMAO as a novel biomarker significantly associated with increased risk of cardiovascular diseases and cancers [3,6]. However, the specific molecular mechanisms underlying TMAO-induced myocardial metabolic abnormalities, its role in cardiovascular disease progression, and the changes produced by MICT intervention remain to be elucidated. This study established a TMAO-induced myocardial injury murine model combined with exercise intervention to systematically investigate TMAO’s impact on cardiac metabolism and exercise-mediated protection. NMR metabolomics revealed that TMAO exposure significantly altered myocardial metabolic profiles, characterized by decreased energy metabolites and abnormal elevations in lipid intermediates. Notably, MICT effectively reversed these metabolic disturbances, which are potentially related to remodeling of energy metabolism and amino acid metabolism, improved mitochondrial function, regulated fatty acid oxidation, and enhanced antioxidant capacity. These findings not only reveal TMAO’s pathogenic role in myocardial metabolic imbalance, but also provide novel theoretical foundations for exercise interventions in preventing and treating cardiovascular metabolic syndromes. However, whether these fundings reflect improved mitochondrial function, altered fatty-acid oxidation, or enhanced antioxidant capacity remains to be tested with direct enzyme-activity or respirometry measurements.

As the organ with the highest energy demand, the heart maintains energy homeostasis by dynamically regulating fatty acid β-oxidation and aerobic glucose metabolism. Elevated blood fatty acid levels trigger preferential reliance on fatty acids for energy production while suppressing glucose utilization [28]. Additionally, ketone bodies and amino acids contribute to myocardial energy metabolism, albeit to a lesser extent. The myocardium exhibits metabolic flexibility, adjusting substrate utilization ratios based on cardiac load, substrate availability, and nutritional status to sustain cardiomyocyte energy supply. Disruption of cardiac metabolic pathways can directly cause structural and functional abnormalities [28,29,30]. Studies indicate that heart failure patients often exhibit impaired glucose uptake alongside hyperactive fatty acid oxidation and compensatory upregulation of ketone metabolism. Our study reveals that TMAO significantly disrupts myocardial energy metabolism: key TCA cycle anaplerotic substrates (aspartate and leucine) decreased significantly, accompanied by reduced myocardial glucose content (Figure S2). Previous studies demonstrate diminished myocardial glucose uptake in murine heart failure models [31], indicating that TMAO-associated changes in TCA-cycle anaplerotic substrates coincide with reduced myocardial glucose content, and suggesting that TMAO may impair mitochondrial energy coupling efficiency, thereby inducing metabolic dysfunction and subsequent structural remodeling. Nevertheless, functional studies (citrate-synthase activity, high-resolution respirometry) are required to determine whether mitochondrial coupling efficiency is altered.

The phenylalanine metabolism pathway exhibited significant abnormalities in TMAO-induced myocardial injury: both phenylalanine and its hydroxylated product tyrosine levels decreased. Studies confirm that phenylacetylglutamine, a metabolite of phenylalanine, associates with cardiovascular disease progression [32,33]. Furthermore, as a catecholamine precursor, tyrosine metabolic disruption may be closely linked to cardiac hypertrophy development [34]. Although prior studies report potential cardioprotective effects of TMAO under certain conditions [35,36], substantial study indicates detrimental impacts of TMAO and its metabolites in cardiovascular pathologies [37]. For instance, TMAO promotes fibroblast-to-myofibroblast transdifferentiation and activates the TGF-β receptor I/Smad 2 pathway, indirectly facilitating fibrosis [38]. This study demonstrates that chronic high-dose TMAO exposure induces pathological alterations including cardiac dysfunction and myocardial hypertrophy, suggesting TMAO’s pathological effects may involve multi-target regulation mediated by its metabolites. This paradoxical evidence necessitates further investigation into the concentration- and duration-dependent biological effects of TMAO.

Alterations in Nicotinate and nicotinamide metabolic pathways suggest TMAO may impair mitochondrial function by disrupting NAD^+^ metabolism. Nicotinate converts to nicotinamide in humans, which constitutes coenzyme I and coenzyme II, participating in lipid metabolism, oxidative processes of tissue respiration, and anaerobic carbohydrate catabolism. Furthermore, NAD^+^ serves as an essential coenzyme for fuel oxidation and oxidative phosphorylation, and as a substrate for enzymatic signaling in energy and oxidative stress responses, emerging as a metabolic target in various diseases [39]. Previous studies demonstrate that nicotinamide riboside effectively restores NAD^+^ synthesis and promotes cardiomyocyte glycolysis, delaying heart failure progression in murine models [40]. Although no concentration changes in Nicotinate/nicotinamide-related metabolites were detected in this study, existing evidence suggests TMAO might suppress NAMPT activity to reduce NAD^+^ levels, consequently compromising cardiac energetics [41]. Notably, exercise improves energy metabolism in heart failure models by upregulating the SIRT1-NAMPT axis [42,43], indicating that targeting this pathway may represent a key strategy for exercise-mediated cardiovascular protection. The lack of data including NAD^+^ concentrations, NAMPT activity, or SIRT1 phosphorylation status makes testing whether TMAO or exercise modulates the NAD^+^ salvage pathway necessary to validate in future study.

Abundant evidence shows that regular aerobic exercise can effectively slow or reverse myocardial injury by improving myocardial energy metabolism, enhancing mitochondrial function, and modulating vascular endothelial function [44,45,46]. Notably, this study found that MICT significantly regulated two metabolic pathways in the myocardium: alanine, aspartate, and glutamate metabolism, and glycine, serine, and threonine metabolism. These essential amino acids are key energy sources for the myocardium, directly or indirectly participating in the TCA cycle [30]. MICT may enhance energy substrate supply to maintain myocardial energy homeostasis. Additionally, the upregulation of glycine, serine, and threonine metabolism not only provides essential substrates for protein synthesis, but may also exert cardioprotective effects through immune modulation [47,48]. Particularly relevant is the established link between dysregulated branched-chain amino acid (BCAA: leucine, isoleucine, valine) metabolism and heart failure pathogenesis [49]. Exercise improves myocardial energy efficiency by activating BCAA catabolic pathways, synergizing with MICT-induced upregulation of glycerolipid metabolism observed herein. Furthermore, as a nitric oxide (NO) precursor, altered arginine metabolism directly impacts NO biosynthesis, exerting significant protective effects against cardiac pathology [50]. Exercise enhances ATPase activity, lactate dehydrogenase activity, and NADH oxidation levels, thereby increasing the myocardial uptake of glucose and fatty acids [51]. Collectively, these findings indicate that MICT establishes a metabolic protective barrier by integrating amino acid (including BCAAs and glycine) and lipid metabolic pathways. This not only alleviates TMAO-induced energetic disruption but also prevents and repairs myocardial injury through enhanced antioxidant capacity and mitochondrial function.

In this study, MICT significantly alleviated high-dose TMAO-induced cardiac injury in mice by modulating tyrosine and purine metabolic pathways. As a precursor of catecholamines, tyrosine improves cardiac function through sympathetic activity regulation and inotropic effects [52]. Heart failure typically involves left ventricular dysfunction and myocardial oxygen consumption imbalance, while purine metabolism enhances ATP generation, improves cardiac energetics/diastolic function, and reduces all-cause mortality in heart-failure patients [53]. Notably, allopurinol—a xanthine oxidase inhibitor—improves cardiac function and exercise tolerance in chronic heart failure patients [54]. This suggests MICT may mitigate TMAO cardiotoxicity by activating tyrosine and purine metabolism. However, studies indicate controversial mortality benefits of purine metabolism modulation in ischemic heart disease patients over 60 without gout [55,56], implying clinical outcomes may depend on patient phenotypes and intervention timing.

This study showed that MICT could effectively alleviate TMAO-induced cardiac injury in mice and revealed its regulatory effects on key metabolic pathways through metabolomics. However, the claims of this study are speculative. All interpretations linking metabolite shifts to mitochondrial performance, NO production, or redox balance remain hypothetical in the absence of enzyme-activity assays, mitochondrial respirometry, or signaling analyses. Future research should combine proteomics and enzyme kinetics experiments to clarify the causal relationship between changes in metabolite levels and myocardial injury repair. Additionally, it is recommended to further explore the synergistic effects of exercise intervention and pharmacological approaches to provide more precise strategies for cardiac metabolic diseases.

## 4. Materials and Methods

### 4.1. Animals and Ethics Statement

Male C57BL/6J mice (11 weeks old, n = 45) were purchased from Beijing Huafukang Biotechnology Co., Ltd. (Beijing, China). All mice were housed in cages at a temperature of 22–24 °C and a relative humidity of 55–70%, with four mice per cage and a 12/12 h light–dark cycle. They had ad libitum access to deionized food and water. All experimental procedures were approved by the Sports Science Experiment Ethics Committee of Beijing Sports University (2021127A).

Forty-five mice were randomly allocated into three groups: the sedentary control group (Con), the TMAO-induced myocardial injury group (TMAO), and the TMAO-induced myocardial injury with MICT intervention group (Exe), with fifteen mice in each group. The Con group did not undergo exercise or TMAO treatment, the TMAO group received TMAO gavage but did not exercise, and the Exe group received TMAO gavage and also underwent exercise intervention.

### 4.2. Determination of the Maximal Running Capacity (MRC) on Treadmill

The MRC test protocol was adapted from the study by Martinez et al. [57]. It started at 6 m/min for 10 min warm-up, and the speed was progressively increased 3 m/min every 3 min until the mice were exhausted. The exhaustion was defined as the point at which the mouse failed to reach the end of the treadmill runway despite gentle tail prodding with a driving stick more than five times. The last speed was defined as the MRC.

### 4.3. Training Protocols

All mice were acclimatized for one week prior to the experiment. Subsequently, they underwent a one-week adaptive exercise training program consisting of five consecutive sessions. The training protocol was as follows: On the first day, mice ran at 6 m/min for 10 min. The speed was incrementally increased by 1 m/min each subsequent day while maintaining the same duration. After completing the adaptive training, mice were allowed a two-day rest period before undergoing the MRC test.

The MICT program was finalized with minor modifications based on previous studies [58,59,60] and preliminary experimental data, which consisted of 5 min warm-up, followed by 50 min of exercise at 50% of MRC, and concluded with a 5 min cool-down period, totaling 60 min. The 50% MRC falls within the ACSM-defined moderate-intensity zone. Although HIIT can induce larger VO_2_ max gains in healthy individuals, direct comparisons in pressure-overload or TMAO models reveal no superior cardio-protection over MICT when total work is matched. Therefore, MICT was chosen as the regimen with the most robust safety record in metabolically stressed hearts. All training sessions were carried out in the afternoon, five times per week for eight weeks. The intensity of MICT was adjusted every two weeks based on the results of biweekly MRC tests.

### 4.4. TMAO Administration

TMAO (Sigma-Aldrich, St. Louis, MO, USA, 95%, 317594-5G) was fully diluted in normal saline (NS) and administered intragastrically (800 mg/kg body weight) daily for eight weeks. Each mouse received 0.3 mL of test solution via oral gavage daily. Control group animals were administered an equivalent volume of NS. All gavage procedures were performed in the morning.

### 4.5. Samples Collection

Twenty-four hours after the final exercise session, cardiac tissue collection was performed. Mice were anesthetized with isoflurane, followed by cardiac functional assessment. Upon completion of functional evaluation, euthanasia was performed. Hearts were then excised, thoroughly rinsed with physiological saline to remove residual blood, and precisely weighed.

### 4.6. Cardiac Functional Assessment in Mice

#### 4.6.1. Echocardiographic Examination

Mice were anesthetized with isoflurane to maintain light surgical anesthesia. Thoracic hair was removed with depilatory cream to ensure acoustic coupling. Mice were positioned in the supine position on a temperature-controlled platform, with limbs gently secured using medical tape to maintain extended positioning. Ultrasound coupling gel was applied to the prepared thoracic region, and cardiac function was evaluated using a portable echocardiography system (VIVID I, GE Healthcare, Chicago, IL, USA) equipped with a 13 MHz transducer. The probe, immersed in coupling gel, was angled at 10–30° relative to the sternum to acquire parasternal long-axis views, followed by M-mode imaging at the papillary muscle level. Heart rate (HR), left ventricular ejection fraction (EF), and fractional shortening (FS) were measured in triplicate during steady-state conditions, with mean values calculated for subsequent statistical analysis.

#### 4.6.2. Myocardial Tissue H&E Staining Protocol

Following tissue collection, cardiac specimens were fixed in 4% paraformaldehyde for 24 h, subsequently dehydrated through a graded ethanol series, and embedded in paraffin. Using a microtome, 5 μm sections were prepared and mounted onto glass slides. Slides underwent sequential processing beginning with baking at 70 °C for 10 min on a slide warmer, followed by dewaxing in two changes of xylene (10 min each). Tissues were then rehydrated through a descending ethanol gradient and rinsed in distilled water. Nuclear staining was performed with hematoxylin (10–30 min at room temperature), followed by differentiation in 1% acid–alcohol and bluing via running tap water rinse (5 min). Cytoplasmic counterstaining was achieved with 0.5% eosin Y solution (1–3 min), followed by mounting using neutral balsam-mediated cover slipping.

### 4.7. NMR Metabolomics Analysis of Mice Myocardial Tissue

#### 4.7.1. Extraction of Mice Myocardial Tissue’s Aqueous Metabolites

Based on our research group’s expertise in NMR metabolomics, aqueous metabolites of the myocardial tissue of mice were extracted for NMR metabolomic analysis. The sample preparation procedure was as follows: First, samples were retrieved from the −80 °C ultra-low temperature freezer and transferred to a 4 °C refrigerator for thawing. After thawing, approximately 50 mg of myocardial tissue was excised and its exact weight recorded. Subsequently, the tissue sample was placed into a 2 mL homogenization tube. Pre-cooled methanol was added at a ratio of 4 mL/g of tissue, followed by the addition of two small magnetic beads according to the manufacturer’s instructions. Homogenization was performed at 65 Hz for 60 s. Next, pre-cooled chloroform and ultrapure water were added at ratios of 4 mL/g and 2.854 mL/g tissue weight, respectively. Homogenization was repeated at 65 Hz for 60 s. Thereafter, the mixture was vortexed for 2 min and centrifuged for 15 min (4 °C, 12,000 rpm). The upper aqueous phase containing methanol and water was aspirated using a pipette and transferred to a new EP tube. The methanol was then evaporated using a nitrogen blowing instrument. The tube was sealed with parafilm punctured with small holes and returned to the −80 °C freezer. Finally, the aqueous solution was lyophilized into solid powder using a freeze dryer.

#### 4.7.2. Preparation for NMR Sample

The resulting solid powder was resuspended in 550 μL of NMR buffer [50 mM PBS, pH 7.4, 10% D_2_O, 1 mM TSP], vortexed thoroughly, and centrifuged for 5 min (4 °C, 2000 rpm). Subsequently, all samples were transferred into 5 mm NMR tubes and centrifuged in an NMR tube low-speed centrifuge (1000 rpm, 5 min).

#### 4.7.3. Measurements of NMR Spectroscopy

All NMR spectra were acquired at 298 K on a Bruker Avance III 850 MHz NMR spectrometer equipped with a TCI cryoprobe (Bruker Bio Spin, Rheinstetten, Germany). The NOESYGPPR1D pulse sequence [RD-G1-90°-tl-90°-τm-G2-90°-ACQ] was employed to obtain one-dimensional (1D) ^1^H spectra. Here, tl denotes a short delay (4 μs), τm represents the mixing time (10 ms), and gradient pulses G1/G2 were applied to enhance water signal suppression. Spectra were collected with a spectral width of 20 ppm and an acquisition time (ACQ) of 1.93 s, accumulating 128 transients into 64k data points.

Metabolite resonances in myocardial tissue aqueous extracts were annotated through comprehensive analysis of 1D ^1^H-NMR spectral data. This process integrated three complementary resources: the Chenomx NMR Suite platform (version 8.3; Chenomx Inc., Edmonton, AB, Canada), the publicly accessible Human Metabolome Database (HMDB; http://www.hmdb.ca, accessed 3 December 2022), and domain-specific literature references.

#### 4.7.4. Preprocess of NMR Spectra

1D ^1^H spectra were preprocessed using MestReNova 9.0 software (Mestrelab Research S.L., Santiago de Compostela, Spain), including phase correction, baseline correction, and TSP referencing. All 1D spectra were then overlaid and peak-aligned sequentially. The water peak region (δ 5.0–4.8) was excised from the spectra to eliminate its influence on spectral integration. The region from δ 9.5 to 0.8 was segmented into bins of 0.001 ppm width, with each bin integrated separately. After data export, integration values of each peak were normalized against the TSP spectral integral using MATLAB R2014b software (The MathWorks, Inc., Natick, MA, USA). Normalized integrals represent relative levels of specified metabolites.

#### 4.7.5. Statistical Analyses of NMR Data

Normalized NMR spectral data were subjected to multivariate analysis using SIMCA-P software (version 14.1.0, MKS Umetrics, Umea AB, Sweden). Data preprocessing employed Pareto scaling to enhance the contribution of low-abundance metabolites. The principal component analysis (PCA) was initially performed to reveal intrinsic metabolic clustering patterns and sample separations. Building upon observed discriminatory trends, supervised partial least squares-discriminant analysis (PLS-DA) was subsequently applied to maximize inter-group separation and refine metabolic pattern recognition. Model robustness was validated through 200 response permutation testing (RPT) cycles. PLS-DA model reliability was confirmed by proximity of R^2^ (goodness-of-fit) and Q^2^ (predictive ability) values approaching 1. Metabolites with variable importance in projection (VIP) scores >1 were identified as significant discriminators.

Meanwhile, a univariate statistical analysis was performed using a one-way ANOVA, followed by post hoc tests to assess group differences. The Tukey’s honestly significant difference (HSD) test was applied to homoscedastic data, while the Games–Howell test was used for heteroscedastic data. All analyses were conducted using SPSS 22.0 software (IBM, New York, NY, USA). Differential metabolites were identified based on an FDR-adjusted q-value of <0.05 (Benjamini–Hochberg method), ensuring statistical significance.

The determination of characteristic metabolites was further conducted through the utilization of dual selection criteria, necessitating an FDR q < 0.05 (from univariate analysis) and VIP > 1 (from PLS-DA). Subsequent analysis of the metabolites was undertaken to ascertain their biological significance and contribution to the observed metabolic differences between the groups.

#### 4.7.6. Metabolic Pathway Analysis

Metabolic pathway analysis was conducted using the MetaboAnalyst 5.0 web platform (https://www.metaboanalyst.ca, accessed on 6 March 2023) to identify significantly dysregulated pathways through a dual-method approach combining metabolite set enrichment analysis (MSEA) and pathway topology analysis (PTA). MSEA identified biologically relevant metabolite sets exhibiting inter-group differential expression, with statistical significance determined at *p* < 0.05. Concurrently, PTA employed relative-betweenness centrality to quantify pathway perturbation magnitude, expressed as pathway impact values. Pathways meeting both significance thresholds (*p* < 0.05 and PIV > 0.1) were classified as substantially altered metabolic signatures.

### 4.8. Statistical Analysis

All experimental data was shown as mean ± SD, and the data analysis was performed using GraphPad Prism software (version 8.3.0., La Jolla, CA, USA). Inter-group differences were assessed by one-way ANOVA, followed by post hoc pairwise comparisons using unpaired (independent) *t*-tests for statistically significant groups. Statistical significance was defined at ns: *p* ≥ 0.05, *: *p* < 0.05, **: *p* < 0.01, ***: *p* < 0.001, ****: *p* < 0.0001.

## 5. Conclusions

Our findings establish, for the first time, a causal link between chronic high-dose TMAO exposure and a distinct cardiac metabolomic signature characterized by depleted TCA-cycle intermediates and perturbed amino-acid homeostasis. Importantly, MICT not only normalized these metabolic derangements but also translated them into measurable improvements in diastolic function and reduced fibrosis. These data provide a robust foundation for several avenues of future research: (i) longitudinal clinical studies that test whether plasma amino-acid ratios (e.g., glutamine/α-ketoglutarate or branched-chain amino acids/TCA-cycle anaplerotic flux) can serve as non-invasive biomarkers of TMAO-related cardiac risk; (ii) integration of high-resolution LC-MS lipidomics and single-cell transcriptomics to capture low-abundance oxylipins and cell-type-specific metabolic reprogramming that may have escaped NMR detection; and (iii) exploration of adjunctive dietary or pharmacological interventions that synergize with exercise to further blunt TMAO toxicity. Collectively, this work positions metabolic reprogramming via MICT as a promising, translatable strategy against gut-microbiota-driven cardiovascular injury.

## Data Availability

The data analyzed during the current study are contained within the article and its Supplementary Materials.

## Supplementary Materials

The following supporting information can be downloaded at https://www.mdpi.com/article/10.3390/ijms26188902/s1.

## References

[1] World Health Organization (2021). Cardiovascular Diseases (CVDs): Key Facts [Internet].

[2] Manor O., Zubair N., Conomos M.P., Xu X., Rohwer J.E., Krafft C.E., Lovejoy J.C., Magis A.T. (2018). A Multi-omic Association Study of Trimethylamine *N*-Oxide. Cell Rep..

[3] Costabile G., Vetrani C., Bozzetto L., Giacco R., Bresciani L., Del Rio D., Vitale M., Della Pepa G., Brighenti F., Riccardi G. (2021). Plasma TMAO increase after healthy diets: Results from 2 randomized controlled trials with dietary fish, polyphenols, and whole-grain cereals. Am. J. Clin. Nutr..

[4] Koay Y.C., Chen Y.-C., A Wali J., Luk A.W.S., Li M., Doma H., Reimark R., Zaldivia M.T.K., Habtom H.T., E Franks A. (2020). Plasma levels of trimethylamine-*N*-oxide can be increased with ‘healthy’ and ‘unhealthy’ diets and do not correlate with the extent of atherosclerosis but with plaque instability. Cardiovasc. Res..

[5] Tang W.W., Wang Z., Kennedy D.J., Wu Y., Buffa J.A., Agatisa-Boyle B., Li X.S., Levison B.S., Hazen S.L. (2015). Gut Microbiota-Dependent Trimethylamine *N*-Oxide (TMAO) Pathway Contributes to Both Development of Renal Insufficiency and Mortality Risk in Chronic Kidney Disease. Circ. Res..

[6] Zeisel S.H., Warrier M. (2017). Trimethylamine *N*-Oxide, the Microbiome, and Heart and Kidney Disease. Annu. Rev. Nutr..

[7] Tang W.H., Wang Z., Fan Y., Levison B., Hazen J.E., Donahue L.M., Wu Y., Hazen S.L. (2014). Prognostic value of elevated levels of intestinal microbe-generated metabolite trimethylamine-*N*-oxide in patients with heart failure: Refining the gut hypothesis. J. Am. Coll. Cardiol..

[8] Wang Z., Klipfell E., Bennett B.J., Koeth R., Levison B.S., DuGar B., Feldstein A.E., Britt E.B., Fu X., Chung Y.-M. (2011). Gut Flora Me-tabolism of Phosphatidylcholine Promotes Cardiovascular Disease. Nature.

[9] Chen M.L., Yi L., Zhang Y., Zhou X., Ran L., Yang J., Zhu J.D., Zhang Q.Y., Mi M.T. (2016). Resveratrol Attenuates Trimethylamine-*N*-Oxide (TMAO)-Induced Atherosclerosis by Regulating TMAO Synthesis and Bile Acid Metabolism via Remodeling of the Gut Microbiota. mBio.

[10] Koeth R.A., Levison B.S., Culley M.K., Buffa J.A., Wang Z.N., Gregory J.C., Org E., Wu Y.P., Li L., Smith J.D. (2014). γ-Butyrobetaine Is a Proatherogenic Intermediate in Gut Microbial Metabolism of L-Carnitine to TMAO. Cell Metab..

[11] Koeth R.A., Wang Z., Levison B.S., Buffa J.A., Org E., Sheehy B.T., Britt E.B., Fu X., Wu Y., Li L. (2013). Intestinal microbiota metabolism of l-carnitine, a nutrient in red meat, promotes atherosclerosis. Nat. Med..

[12] Tan X., Liu Y., Long J., Chen S., Liao G., Wu S., Li C., Wang L., Ling W., Zhu H. (2019). Trimethylamine *N*-Oxide Aggravates Liver Steatosis through Modulation of Bile Acid Metabolism and Inhibition of Farnesoid X Receptor Signaling in Nonalcoholic Fatty Liver Disease. Mol. Nutr. Food Res..

[13] Bjornestad E.O., Dhar I., Svingen G.F.T., Pedersen E.R., Orn S., Svenningsson M.M., Tell G.S., Ueland P.M., Sulo G., Laaksonen R. (2022). Circulating trimethylamine *N*-oxide levels do not predict 10-year survival in patients with or without coronary heart disease. J. Intern. Med..

[14] Zhang X., Li Y., Yang P., Liu X., Lu L., Chen Y., Zhong X., Li Z., Liu H., Ou C. (2020). Trimethylamine-*N*-Oxide Promotes Vascular Calcification Through Activation of NLRP3 (Nucleotide-Binding Domain, Leucine-Rich-Containing Family, Pyrin Domain-Containing-3) Inflammasome and NF-κB (Nuclear Factor κB) Signals. Arter. Thromb. Vasc. Biol..

[15] Kong Q., Gu J., Lu R., Huang C., Chen L., Wu W., Lin D. (2022). NMR-Based Metabolomic Analysis of Cardiac Tissues Clarifies Molecular Mechanisms of CVB3-Induced Viral Myocarditis and Dilated Cardiomyopathy. Molecules.

[16] Engel L.E., de Souza F.L.A., Giometti I.C., Okoshi K., Mariano T.B., Ferreira N.Z., Pinheiro D.G., Floriano R.S., Aguiar A.F., Cicogna A.C. (2022). The high-intensity interval training mitigates the cardiac remodeling in spontaneously hypertensive rats. Life Sci..

[17] Frasier C.R., Moore R.L., Brown D.A. (2011). Exercise-induced cardiac preconditioning: How exercise protects your achy-breaky heart. J. Appl. Physiol..

[18] Powers S.K., Quindry J.C., Kavazis A.N. (2008). Exercise-induced cardioprotection against myocardial ischemia–reperfusion injury. Free. Radic. Biol. Med..

[19] Padrao A.I., Moreira-Gonçalves D., Oliveira P.A., Teixeira C., Faustino-Rocha A.I., Helguero L., Vitorino R., Santos L.L., Amado F., Duarte J.A. (2015). Endurance training prevents TWEAK but not myostatin-mediated cardiac remodelling in cancer cachexia. Arch. Biochem. Biophys..

[20] Pagan L.U., Gomes M.J., Damatto R.L., Lima A.R.R., Cezar M.D.M., Damatto F.C., Reyes D.R.A., Campos D.H.S., Caldonazo T.M.M., Polegato B.F. (2021). Aerobic Exercise During Advance Stage of Uncontrolled Arterial Hypertension. Front. Physiol..

[21] Miyachi M., Yazawa H., Furukawa M., Tsuboi K., Ohtake M., Nishizawa T., Hashimoto K., Yokoi T., Kojima T., Murate T. (2009). Exercise Training Alters Left Ventricular Geometry and Attenuates Heart Failure in Dahl Salt-Sensitive Hypertensive Rats. Hypertension.

[22] Chacko S., Haseeb Y.B., Haseeb S. (2022). Metabolomics Work Flow and Analytics in Systems Biology. Curr. Mol. Med..

[23] Gowda G.A.N., Raftery D. (2023). NMR Metabolomics Methods for Investigating Disease. Anal. Chem..

[24] Gowda G.A.N., Zhu W., Raftery D. (2025). NMR-based metabolomics: Where are we now and where are we going?. Prog. Nucl. Magn. Reson. Spectrosc..

[25] Emwas A.-H.M., Salek R.M., Griffin J.L., Merzaban J. (2013). NMR-based metabolomics in human disease diagnosis: Applications, limitations, and recommendations. Metabolomics.

[26] Bansal T., Alaniz R.C., Wood T.K., Jayaraman A. (2010). The bacterial signal indole increases epithelial-cell tight-junction resistance and attenuates indicators of inflammation. Proc. Natl. Acad. Sci. USA.

[27] Bravo J.A., Forsythe P., Chew M.V., Escaravage E., Savignac H.M., Dinan T.G., Bienenstock J., Cryan J.F. (2011). Ingestion of Lactobacillus strain regulates emotional behavior and central GABA receptor expression in a mouse via the vagus nerve. Proc. Natl. Acad. Sci. USA.

[28] Doenst T., Bugger H., Schwarzer M., Faerber G., Borger M.A., Mohr F.W. (2008). Three good reasons for heart surgeons to understand cardiac metabolism. Eur. J. Cardio Thorac. Surg..

[29] Li X., Wu F., Günther S., Looso M., Kuenne C., Zhang T., Wiesnet M., Klatt S., Zukunft S., Fleming I. (2023). Inhibition of fatty acid oxidation enables heart regeneration in adult mice. Nature.

[30] Lopaschuk G.D., Karwi Q.G., Tian R., Wende A.R., Abel E.D. (2021). Cardiac Energy Metabolism in Heart Failure. Circ. Res..

[31] Bei Y., Zhu Y., Zhou J., Ai S., Yao J., Yin M., Hu M., Qi W., Spanos M., Li L. (2024). Inhibition of Hmbox1 Promotes Cardiomyocyte Survival and Glucose Metabolism Through Gck Activation in Ischemia/Reperfusion Injury. Circulation.

[32] Nemet I., Saha P.P., Gupta N., Zhu W., Romano K.A., Skye S.M., Cajka T., Mohan M.L., Li L., Wu Y. (2020). A Cardiovascular Disease-Linked Gut Microbial Metabolite Acts via Adrenergic Receptors. Cell.

[33] Hanley W.B. (2013). Optimal serum phenylalanine for adult patients with phenylketonuria (PKU). Mol. Genet. Metab..

[34] Xu M.G., Bermea K.C., Ayati M., Kim H.B., Yang X.M., Medina A., Fu Z.M., Heravi A., Zhang X.Y., Na C.H. (2022). Alteration in tyrosine phosphorylation of cardiac proteome and EGFR pathway contribute to hypertrophic cardiomyopathy. Commun. Biol..

[35] Melita V., Reinis V., Stanislava K., Helena C., Eduards S., Maija D., Marina M.-K. (2021). Microbiota-Derived Metabolite Trimethylamine *N*-Oxide Protects Mitochondrial Energy Metabolism and Cardiac Functionality in a Rat Model of Right Ventricle Heart Failure. Front. Cell Dev. Biol..

[36] Tomasz H., Adrian D., Marta G., Marek K., Klaudia B., Ewelina Z., Emilia S., Aleksandra W.-T., Leszek P., Michal D. (2018). Chronic, low-dose TMAO treatment reduces diastolic dysfunction and heart fibrosis in hypertensive rats. Am. J. Physiol. Heart Circ. Physiol..

[37] Luqman A., Hassan A., Ullah M., Naseem S., Ullah M., Zhang L., Din A.U., Ullah K., Ahmad W., Wang G. (2024). Role of the intestinal microbiome and its therapeutic intervention in cardiovascular disorder. Front. Immunol..

[38] Wenlong Y., Shuning Z., Jianbing Z., Hao J., Daile J., Tiantong O., Zhiyong Q., Yunzeng Z., Juying Q., Aijun S. (2019). Gut microbe-derived metabolite trimethylamine *N*-oxide accelerates fibroblast-myofibroblast differentiation and induces cardiac fibrosis. J. Mol. Cell. Cardiol..

[39] Zapata-Perez R., Wanders R.J.A., van Karnebeek C.D.M., Houtkooper R.H. (2021). NAD(+) homeostasis in human health and disease. EMBO Mol. Med..

[40] Diguet N., Trammell S.A.J., Tannous C., Deloux R., Piquereau J., Mougenot N., Gouge A., Gressette M., Manoury B., Blanc J. (2018). Nicotinamide Riboside Preserves Cardiac Function in a Mouse Model of Dilated Cardiomyopathy. Circulation.

[41] Hara N., Osago H., Hiyoshi M., Kobayashi-Miura M., Tsuchiya M. (2019). Quantitative analysis of the effects of nicotinamide phosphoribosyltransferase induction on the rates of NAD+ synthesis and breakdown in mammalian cells using stable isotope-labeling combined with mass spectrometry. PLoS ONE.

[42] Lv J., Li Y., Shi S., Xu X., Wu H., Zhang B., Song Q. (2022). Skeletal muscle mitochondrial remodeling in heart failure: An update on mechanisms and therapeutic opportunities. Biomed. Pharmacother..

[43] de Souza S.L.B., Mota G.A.F., da Silva V.L., Vileigas D.F., Sant’ANa P.G., Gregolin C.S., Figueira R.L., Batah S.S., Fabro A.T., Murata G.M. (2023). Effects of early exercise on cardiac function and lipid metabolism pathway in heart failure. J. Cell. Mol. Med..

[44] Zhou Q., Deng J., Pan X., Meng D., Zhu Y., Bai Y., Shi C., Duan Y., Wang T., Li X. (2022). Gut microbiome mediates the protective effects of exercise after myocardial infarction. Microbiome.

[45] Valenzuela P.L., Ruilope L.M., Santos-Lozano A., Wilhelm M., Kraenkel N., Fiuza-Luces C., Lucia A. (2023). Exercise benefits in cardiovascular diseases: From mechanisms to clinical implementation. Eur. Heart J..

[46] Hou S.L., Liu L., Yao J., Zhao Q., Feng W., Liu Q.Q., Zou M., Zhang R.X., Yin H.T., Xian H.M. (2025). Impact of exercise-based cardiac rehabilitation on cardiopulmonary function and prognosis in ST elevation myocardial infarction after PCI patients in extremely cold regions. BMC Cardiovasc. Disord..

[47] Warnecke G., Schulze B., Steinkamp T., Haverich A., Klima U. (2006). Glycine application and right heart function in a porcine heart transplantation model. Transpl. Int..

[48] Zhang Y., Su W., Zhang Q., Xu J., Liu H., Luo J., Zhan L., Xia Z., Lei S. (2018). Glycine Protects H9C2 Cardiomyocytes from High Glucose- and Hypoxia/Reoxygenation-Induced Injury via Inhibiting PKCbeta2 Activation and Improving Mitochondrial Quality. J. Diabetes Res..

[49] Wang M., Liu Z., Ren S., Zhu J., Morisawa N., Chua G.L., Zhang X., Wong Y.K., Su L., Wong M.X. (2025). BCAA catabolism targeted therapy for heart failure with preserved ejection fraction. Theranostics.

[50] Farah C., Michel L.Y.M., Balligand J.L. (2018). Nitric oxide signalling in cardiovascular health and disease. Nat. Rev. Cardiol..

[51] Ferreira R., Nogueira-Ferreira R., Trindade F., Vitorino R., Powers S.K., Moreira-Goncalves D. (2018). Sugar or fat: The metabolic choice of the trained heart. Metabolism.

[52] Saito Y., Sugiura Y., Sakaguchi A., Sada T., Nishiyama C., Maeda R., Kaneko M., Kiyonari H., Kimura W. (2024). Redox-dependent purine degradation triggers postnatal loss of cardiac regeneration potential. Redox Biol..

[53] Manzoni A.G., Passos D.F., Doleski P.H., Leitemperger J.W., Loro V.L., Leal D.B.R. (2020). Purine Metabolism in Platelets and Heart Cells of Hyperlipidemic Rats. Cardiovasc. Drugs Ther..

[54] Alem M.M., Alshehri A.M., Cahusac P.M., Walters M.R. (2018). Effect of Xanthine Oxidase Inhibition on Arterial Stiffness in Patients With Chronic Heart Failure. Clin. Med. Insights Cardiol..

[55] Mackenzie I.S., Hawkey C.J., Ford I., Greenlaw N., Pigazzani F., Rogers A., Struthers A.D., Begg A.G., Wei L., Avery A.J. (2022). Allopurinol versus usual care in UK patients with ischaemic heart disease (ALL-HEART): A multicentre, prospective, randomised, open-label, blinded-endpoint trial. Lancet.

[56] Tanaka A., Node K. (2022). Xanthine oxidase inhibition for cardiovascular disease prevention. Lancet.

[57] Martinez-Huenchullan S.F., Ban L.A., Olaya-Agudo L.F., Maharjan B.R., Williams P.F., Tam C.S., Mclennan S.V., Twigg S.M. (2019). Constant-Moderate and High-Intensity Interval Training Have Differential Benefits on Insulin Sensitive Tissues in High-Fat Fed Mice. Front. Physiol..

[58] Baekkerud F.H., Salerno S., Ceriotti P., Morland C., Storm-Mathisen J., Bergersen L.H., Hoydal M.A., Catalucci D., Stolen T.O. (2019). High Intensity Interval Training Ameliorates Mitochondrial Dysfunction in the Left Ventricle of Mice with Type 2 Diabetes. Cardiovasc. Toxicol..

[59] Kemi O.J., Loennechen J.P., Wisloff U., Ellingsen O. (2002). Intensity-controlled treadmill running in mice: Cardiac and skeletal muscle hypertrophy. J. Appl. Physiol..

[60] Chavanelle V., Boisseau N., Otero Y.F., Combaret L., Dardevet D., Montaurier C., Delcros G., Peltier S.L., Sirvent P. (2017). Effects of high-intensity interval training and moderate-intensity continuous training on glycaemic control and skeletal muscle mitochondrial function in db/db mice. Sci. Rep..

