# Supplementary material for: The Protective Mechanism of Moderate Intensity Continuous Training on TMAO-Induced Myocardial Injury Based on NMR Metabolomics
Source: Int J Mol Sci. 2025 Sep 12;26(18):8902. doi: 10.3390/ijms26188902 (PMC12470193; doi:10.3390/ijms26188902)
Supplement: Supplementary file 1 [file ijms-26-08902-s001.zip › ijms-3797695-supplementary.pdf]

Supplementary materials

Figure S1.

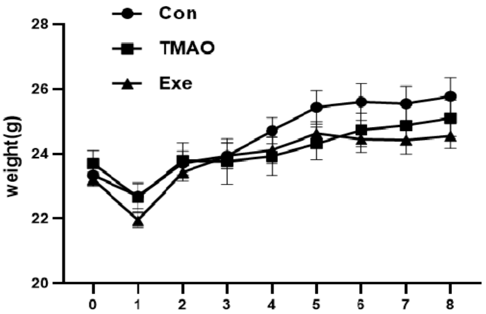

Figure S1. Weight change curve of three groups of mice.

Figure S2.

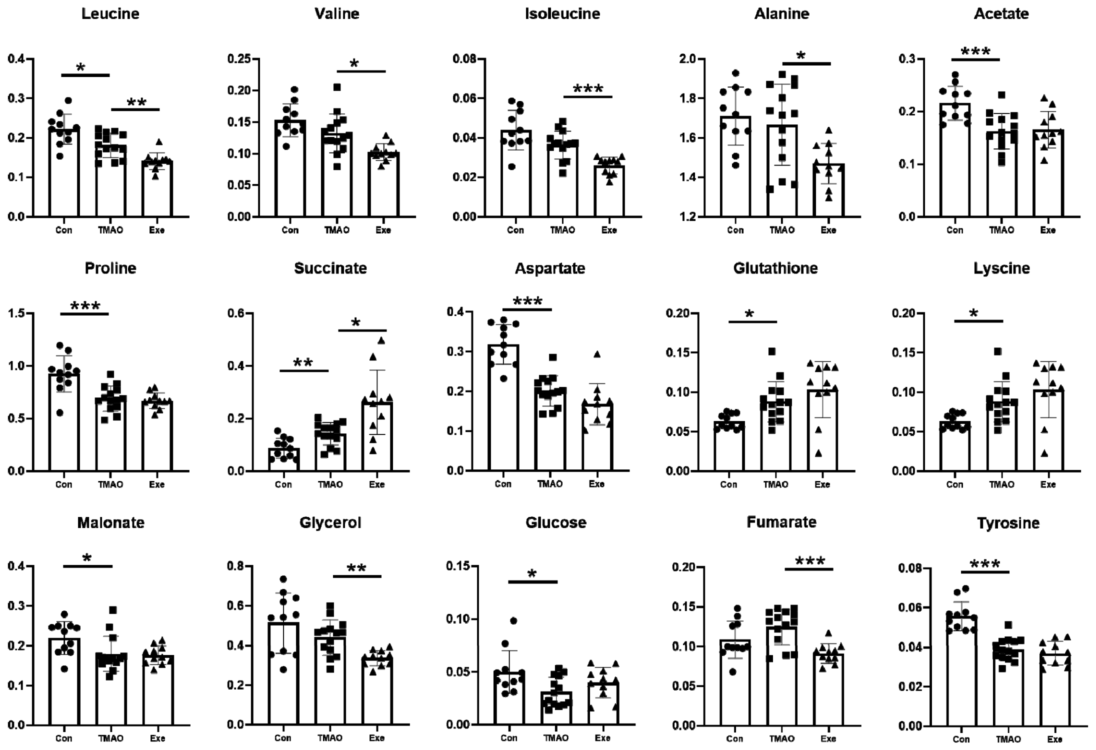

Figure S2. Concentration analysis of metabolite levels between the three groups of the myocardial tissue of mice based on relative NMR integrals. Data are expressed as mean ± SD. One-way ANOVA with Benjamini-Hochberg correction was used to determine significant changes in metabolite levels between groups. Statistical significance is indicated as follows: * FDR $q < 0.05$, **FDR $q < 0.01$, ***FDR $q < 0.001$, ****FDR $q < 0.0001$.

Supplementary Tables

Table S1. Identified metabolites in ^{1}H NMR spectra of aqueous extracts derived from the myocardial tissue in three groups of mice.

NO.	Metabolites	δ ^{1}H (ppm) and multiplicity	Moieties
1	Pantothenate	0.88(s), 0.92(s)	CH_3, CH_3
2	Leucine	0.96(d), 0.97(d), 1.69(m), 1.70(m), 1.73(m), 3.73(m)	α-CH_3, α-CH_3, γ-CH, β-CH_2, α-CH
3	Valine	0.99(d), 1.05(d), 2.26(m), 3.60(d)	γ-CH_3, γ-CH_3, β-CH, α-CH
4	Isoleucine	0.94(t), 1.01(d), 1.21(m), 1.42(m), 2.00(m), 3.67(d)	δ-CH_3, γ-CH_3, half γ-CH_2, half γ-CH_2, β-CH, α-CH
5	Methylsuccinate	2.61(m), 2.51(dd),1.07(d)	CH, CH2, CH_3
6	Ethanol	1.17(t), 3.65(q)	δ-CH_3, CH_2
7	3-Hydroxybutyrate	1.20(d), 2.30(q), 2.39(q), 4.14(m)	γ-$CH_{2,}$ β-CH_2, γ-CH
8	lactate	1.33(d), 4.11(q)	β-CH_3, α-CH
9	Alanine	1..47(d), 3.78(q)	β-CH_3, α-CH $_2$
10	Acetate	1.91(s)	CH_3
11	Proline	1.99(m)	γ-CH_2
12	Glutamate	2.08(m), 2.12(m), 2.34(m), 2.37(m), 3.75(m)	half β-CH_2, half β-CH_2, half γ-CH_2, half γ-CH_2, α-CH
13	Glutamine	2.13(m), 2.45(m), 3.77(t)	γ-CH_2, β-CH_2, α-CH
14	Pyruvate	2.41(s)	α-CH_3
15	Succinate	2.41(s)	CH
16	Aspartate	2.68(dd), 2.81(dd), 3.90(dd)	β-CH_2, α-CH
17	Asparagine	2.84(dd), 2.94(dd), 4.00(dd)	half β-CH, half β-CH, α-CH
18	Trimethylamine	2.88(s)	CH_3
19	Glutathione	2.15(m), 2.55(m), 2.96(m), 3.77(m), 4.56(m)	β-CH_2, γ-CH_2, CH_2-SH, α-CH&CH_2-NH, CH-NH
20	Lysine	1.43(m), 1.50(m), 1.73(m), 1.89(m), 1.92(m), 3.02(t), 3.75(t)	γ-CH_2, half γ-CH_2, δ-CH_2, β-CH_2, ε-CH_2, α-CH
21	Creatine	3.04(s), 3.93(s)	N-CH_3, α-CH_2
22	Malonate	3.11(s)	CH_2
23	Choline	3.20(s), 3.50 (dd), 4.03(t)	N-$(CH_3)_3$, N-CH_2, CH_2OH
24	O-Phosphocholine	3.22(s), 3.59(m),4.17(m)	CH_3, N-CH2, O-CH2
25	Sn-Glycero-3-phosphocholine	3.23(s), 3.60(dd), 3.68(dd), 3.87(m), 3.94(m), 4.33(m)	N-$(CH_3)_3$, half $^1CH_2$, $^2CH_2$, half $^1CH_2$, half $^3CH_2$, half $^3CH_2$, $^1CH_2$
26	carnitine	3.21(s)	CH_3
27	β-Alanine	2.54(t), 3.17(t)	CH_2, CH_2

28	TMAO		
29	Methanol	3.34(s)	CH_3
30	Taurine	3.24(t), 3.41(t)	$^{1}CH_2$, $^{2}CH_2$
31	Myo-Inositol	3.28(t), 3.53 (dd), 3.63(t), 4.07(t)	^{2}CH, $^{4,6}CH$, $^{1,3}CH$, ^{5}CH
32	Glycine	3.57(s)	α-CH_2
33	Glycerol	3.98 (M), 4.33 (m)	NH_2, δ-CH,
34	Glucose	β(3.24 (dd), 3.48(t), 3.90 (dd)), α(3.54 (dd), 3.71(t), 3.72 (dd), 3.83(m))	β(H2, H3, H5), α(H2, H3, H6)
35	Fumarate	8.46(s)	CH
36	Tyrosine	3.05(dd), 3.19(dd), 6.92(d), 7.19(d)	half β-CH_2, half β-CH_2, β-CH, α-CH
37	Phenylalanine	3.12(dd), 3.30(dd), 3.99(dd), 7.33(d), 7.37(t), 7.43(t)	α-CH, half β-CH_2, half β-CH_2, α-CH, β-CH, γ-CH
38	Inosine	6.09(d), 8.22(s), 8.34(s)	CH(2), N-CH=N
39	Formate	8.46(s)	CH
40	IMP	4.02(m),4.37(m),4.52(q),6.15(d),8.23(s),8.58(s)	CH_2, CH, CH', N-CH-O, N=CH-N. N=CH
41	ADP	6.13(d),8.27(s),8.58(s)	NH_2, δ-CH, CH (2)
42	AMP	4.51(m), 6.14(d), 8.26(s), 8.62(s)	CH-OH, CH-N, N-CH=N, N-CH=N
43	Niacinamide	7.60(dd),8.23(dd),8.70(dd),8.92(s)	α-CH, β-CH, N=CH, N-CH

Table S2.

Table S2. Differential metabolites of aqueous extracts derived from the myocardial tissue in the TMAO vs. Con and the Exe vs. TMAO.

Differential metabolites	TMAO vs. Con	Exe vs. TMAO
Leucine	√	√
Valine		√
Isoleucine		√
Alanine		√
Acetate	√	
Proline	√	
Succinate	√	√
Aspartate	√	
Glutathione	√	
Lysine	√	
Malonate	√	
Glycerol		√
Glucose	√	
Fumarate		√
Tyrosine	√	
